# Pathogenic, or Toxic Effect of Tobacco

**Published:** 1893-04

**Authors:** S. Seward

**Affiliations:** Syracuse, N. Y.


### PATHOGENETIC, OR TOXIC EFFECT OF TOBACCO.

S. Seward, M. D., Syracuse, N. Y.

The first severe case of tobacco poisoning I ever treated was in a woman, from smoking a clay pipe. Her tongue was coated as with thick, white velvet, and had been so eight years, from a severe sickness. No red color on the tongue or its edges. She smoked almost constantly day and night. There came a crease across the middle of her tongue, as though made with the edge of a knife, and in the centre of the crease there came up an oval point, which increased quite fast until it became as large as the cup of an acorn from an oak, the oval up, then it fell in and disappeared, leaving a cavity covered with a substance appearing like cigar ashes. The tongue was gradually all eaten away into the throat. She suffered severe pain for months, emaciated, and died.

The next severe case I met with and treated was a physician, who had smoked for nearly fifty years. His worst suffering was in the stomach. He had a "sinking sensation in the stomach which obliged him to lie down," which is both pathogenetic and clinical. He would suffer with the sinking sensation for several hours, then it would pass away. He was weak in body and mind. Constipation was the next most severe trouble; there was a loss of power in the colon and rectum to pass the fæces downward, one to three or four injections of warm water would not always bring a stool. He had suffered in this way for several years, becoming worse, and died. On *post mortem*, a malignant tumor was found back of the stomach, and attached to the spine and one kidney, which was doubtless caused by tobacco.

An English workingman came to me with a swelling on his under lip as large as half a butternut, on the place where he rested his clay pipe. I told him it was a tobacco cancer, and could not be cured. He replied, "So all the doctors say." He died in consequence of the accumulated poison of tobacco.

A man with a good constitution and otherwise of good habits, except in the use of tobacco; had been in a business office for about forty years, where himself or some one else was smoking nearly all the time. His whole body suffered, he was bloated and dropsical—the abdomen the most so—face of an earthly-pale color; his liver was enlarged, not sensitive; the urine half albumen. Left side of head was numb, ear deaf, tongue foul, and taste bad, no appetite, quite weak. Many of these symptoms were removed by Apis—the albumen from the urine and the dropsy. Of course he left off the use of tobacco. He was quite well for over four years. He then thought he would like to taste tobacco. He took a little in his mouth. It soon awoke all the old tobacco symptoms, and they could not be removed; the force of the renewed trouble was in the abdomen, and the efforts and wisdom of four physicians could not remove the very painful disease. One of our eclectic homœopaths cut the Gordian knot by a dose of Morphine, which soon ended his sufferings and life.

This patient, a man, smoked excessively, and was up nights, a gambler. He was paralyzed on the right side—nervous apoplexy; his expression of a thought was obstructed; he could only speak the first word of the sentence he had in mind, and after a few days he could only speak the first letter of the first word. He improved under treatment, and went to business again. I cautioned him to keep clear of tobacco, not even to smell it. He felt so well be did not believe it would hurt him to smoke a cigar occasionally. He did so, and was paralyzed, and died suddenly.

